# Crystal structure and Hirshfeld surface analysis of ((*S*,*S*)-2,2′-{[(1,2-di­phenyl­ethane-1,2-di­yl)bis­[(aza­n­ium­ylyl­idene)methanylyl­idene]}bis­(6-meth­oxy­phenolato))trinitratosamarium(III)

**DOI:** 10.1107/S2056989021004424

**Published:** 2021-04-30

**Authors:** Yuta Okumura, Yuji Takiguchi, Daisuke Nakane, Takashiro Akitsu

**Affiliations:** a Department of Chemistry, Faculty of Science, Tokyo University of Science, 1-3 Kagurazaka, Shinjuku-ku, Tokyo 162-8601, Japan

**Keywords:** Schiff base ligand, samarium, chirality, Hirshfeld analysis, crystal structure

## Abstract

In the title complex, [Sm(NO_3_)_3_(C_30_H_28_N_2_O_4_)], the Sm atom is surrounded by ten O atoms. The (*S*,*S*)-2,2′-{[(1,2-di­phenyl­ethane-1,2-di­yl)bis­[(aza­niumylyl­idene)methanylyl­idene]}bis­(6-meth­oxy­phenolate) ligand, obtained from *o*-vanillin and (1*S*,2*S*)-(−)-1,2-di­phenyl­ethyl­enedi­amine, exhibits a slightly distorted planar arrangement of the four coordinated O atoms. In the crystal, the complex shows intra­molecular N—H⋯O hydrogen bonds and weak inter­molecular C—H⋯O hydrogen bonds. The Hirshfeld surface analysis indicates that the most important contributions to the packing are from H⋯H (33.5%), O⋯H (34.1%) and C⋯H (21.7%) contacts.

## Abstract 2

The asymmetric unit of the title mononuclear ten-coordinated samarium chiral Schiff base complex prepared from *o-*vanillin, (1*S*,2*S*)-(−)-1,2-di­phenyl­ethyl­enedi­amine and samarium nitrate hexa­hydrate contains two crystallographically independent mol­ecules.

## Chemical context   

Lanthanide metal complexes can have attractive functions such as magnetism and fluorescence when synthesized with properly designed ligands (Yao *et al.*, 2019 ▸; Lin *et al.*, 2009 ▸). In recent years, lanthanide complexes that act as single-mol­ecule magnets (SMM) have received much attention (Then *et al.*, 2015 ▸). In these complexes, distortion of the coordination geometry is an important factor for magnetic anisotropy and for the resulting SMM properties. However, the coordination chemistry of lanthanides is complicated, and it is necessary to prepare complexes with coordination environments suitable for the required properties. On the other hand, salen ligands are known to form stable chelate complexes with many metals (Cozzi *et al.*, 2004 ▸). By incorporating a substituent group into salen ligands, it is possible to easily add more coordination sites and optical functionality such as the antenna effect that depend on inter­molecular inter­actions and arrangements. Hence, functional lanthanide salen complexes have attracted attention (Ren *et al.*, 2016 ▸). Accurate data such as bond angles and the geometry of coordination sites obtained based on crystal structure analysis and Hirshfeld surface analysis will be useful for the mol­ecular design of new lanthanide and salen complexes. In this study, we prepared a new Sm^III^–salen complex and report herein on its crystal structure and Hirshfeld surface analysis.

## Structural commentary   

The title Sm^III^ complex crystallizes in the monoclinic space group *C*2. The asymmetric unit contains two crystallographically independent mol­ecules. This distorted prismatic [SmO_10_] complex consists of three bidentate nitrate ions and two pairs of phenolate and meth­oxy groups of the salen ligand, which is slightly distorted from planar.
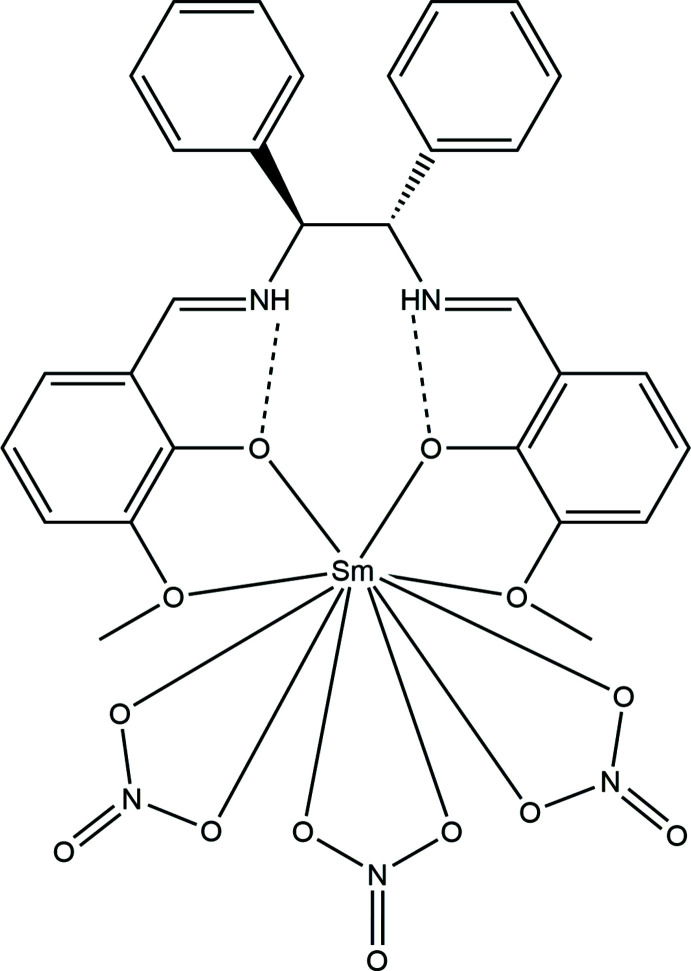


The bond distances between the metal center and ligating atoms range from 2.333 (5) to 2.373 (4) Å for the phenolato oxygen atoms, and from 2.606 (5) to 2.621 (6) Å for meth­oxy oxygen atoms. The bond lengths between the metal center and the nitrate oxygen atoms range from 2.475 (5) to 2.633 (5) Å, showing more flexibility than those of the Schiff base ligand. In the Schiff base ligand, the imine moieties are protonated to form iminium cations, but the C=N bond lengths remain close to those of normal imine bonds at 1.287 (8) and 1.30 (1) Å.

**Figure 1:**
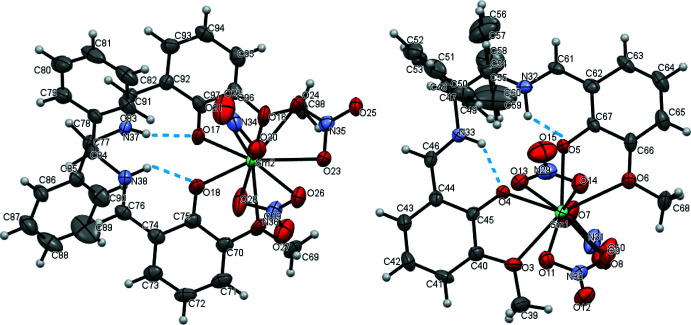
View of the two independent complex mol­ecules of the title compound, showing the atom-labelling scheme. Displacement ellipsoids are drawn at the 30% probability level. Intra­molecular hydrogen bonds are shown as dashed lines. All non-H atoms should be labelled

Intra­molecular hydrogen bonds occur between the iminium protons and the phenolic oxygen atoms, with lengths of 1.71–1.89 Å (Table 1 ▸, Fig. 1 ▸). The bond distances and angles in the ligand are similar to those of analogous complexes (Hayashi *et al.*, 2013 ▸).

## Supra­molecular features   

**Figure 2:**
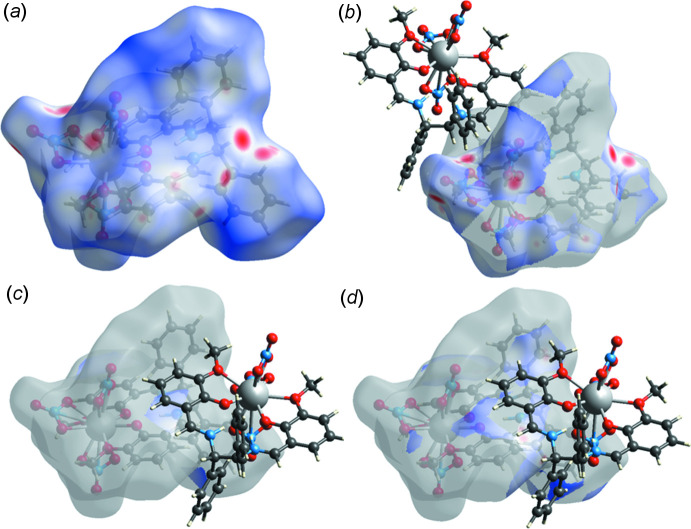
Hirshfeld surfaces plotted over of *d*_norm for_ (*a*) all inter­actions and (*b*) O⋯H/H⋯O, (*c*) C⋯C and (*d*) C⋯H/H⋯C contacts.

**Figure 3:**
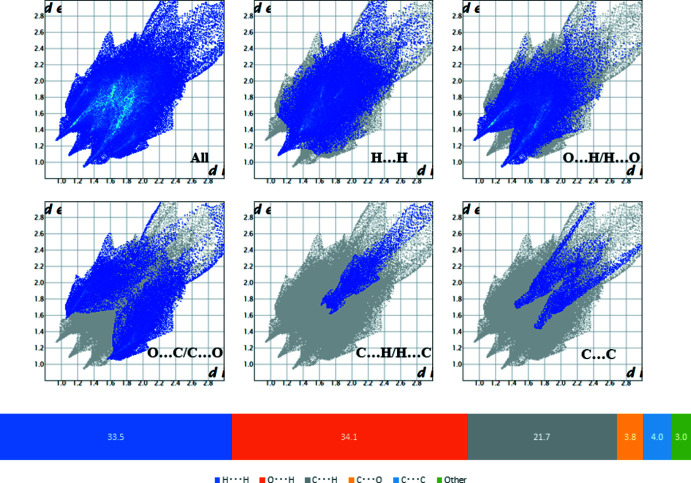
Two-dimensional fingerprint plots and contributions for various inter­actions.

Though some weak C—H⋯O inter­molecular inter­actions are found (Table 1 ▸), no strong inter­actions such as O—H⋯O hydrogen bonds between mol­ecules are observed in the crystal. Hirshfeld surface analysis (Spackman *et al.*, 2009 ▸) was performed to investigate inter­actions in the crystal packing. Hirshfeld surfaces and fingerprint plots (McKinnon *et al.*, 2004 ▸) were calculated using *CrystalExplorer17.5* (Turner *et al.*, 2017 ▸). Hydrogen bonds are strong inter­actions and they are indicated as red dots on the surface (Fig. 2 ▸) or two sharp spikes in the fingerprint plot (Fig. 3 ▸). ‘Wings’ in the fingerprint plots and diagonal plots at 1.8 Å are regarded as a characteristic feature potentially resulting from aromatic rings (Spackman *et al.*, 2002 ▸)The contributions to the Hirshfeld surface are H⋯H (33.5%), O⋯H (34.1%) and C⋯H (21.7%) contacts.

## Database survey   

A search in the Cambridge Structural Database (CSD, Version 5.41, update of November 2019; Groom *et al.*, 2016 ▸) for similar structures returned two relevant entries: (*N*,*N*′-ethane-1,2-diylbis{[2-(­oxy)-3-(meth­oxy)phen­yl]methaniminiumato})tris(nitrato)samarium (refcode MOLNEI; Yang *et al.*, 2013 ▸) and (*S*,*S*)-{*μ*-[2,2′-{(1,2-di­phenyl­ethane-1,2-di­yl)bis­[(aza­nylyl­idene)methylyl­idene]}bis[6-(meth­oxy)phenolato]]}trinitratoeuropium(III)nickel(II) (JIWNEL; Mayans *et al.*, 2019 ▸). In MOLNEI, a similar intra­molecular N—H⋯O hydrogen bond is observed. Although the ligand of JIWNEL is similar to that in the title compound, the coordinating sites are filled with europium(III) and nickel(II) ions. For both MOLNEI and JIWNEL, the crystal packing is dominated by van der Waals inter­actions and C—H⋯O hydrogen bonds.

## Synthesis and crystallization   

(1*S*,2*S*)-(−)-1,2-Di­phenyl­ethyl­enedi­amine (0.100 g, 0.471 mmol) and *o*-vanillin (0.143 g, 0.940 mmol) were dissolved in ethanol (30 mL) and the resulting mixture was stirred at 313 K for 1 h to afford a yellow solution. To this solution, samarium nitrate hexa­hydrate (0.208 g, 0.468 mmol) was added and it was stirred at 313 K for 2 h. A yellow precipitate appeared immediately. The precipitate was filtered and washed with ethanol and hexane. The title compound (0.299 g, 0.366 mmol, yield 78.2%) was obtained as a yellow solid. IR (KBr, cm^−1^) : 1624 (C=N double bond). Fluorescence bands in methanol solution were observed at 562 (^4^G_5/2_ → ^6^H_5/2_), 597 (^4^G_5/2_ → ^6^H_7/2_) and 644 (^4^G_5/2_ → ^6^H _9/2_) nm. Single crystals suitable for X-ray diffraction were obtained by recrystallization from methanol and diethyl ether (1:4, *v*/*v*) solution.

## Refinement   

Crystal data, data collection and structure refinement details are summarized in Table 2 ▸. All C-bound H atoms were placed in geometrically calculated positions (C—H = 0.93–0.98 Å) and were constrained using a riding model with *U*_iso_(H) = 1.2*U*_eq_(C) or 1.5*U*_eq_(C-meth­yl). SIMU, ISOR and AFIX 66 commands were used for C55, C56, C57, C58, C59, C60 to suppress temperature anisotropy and restrain bond lengths to appropriate values.

## Supplementary Material

Crystal structure: contains datablock(s) 1R, I. DOI: 10.1107/S2056989021004424/tx2039sup1.cif

Structure factors: contains datablock(s) I. DOI: 10.1107/S2056989021004424/tx2039Isup2.hkl

CCDC reference: 2080014

Additional supporting information:  crystallographic information; 3D view; checkCIF report

## Figures and Tables

**Table 1:** Hydrogen-bond geometry (Å, °)

*D*—H⋯*A*	*D*—H	H⋯*A*	*D*⋯*A*	*D*—H⋯*A*
C98—H98*A*⋯O9^i^	0.98	2.58	3.419 (9)	144
C91—H91⋯O27^ii^	0.95	2.59	3.097 (8)	114
C91—H91⋯O12^iii^	0.95	2.33	3.227 (8)	156
C77—H77⋯O24^iv^	1.00	2.29	3.264 (8)	164
C76—H76⋯O21^iv^	0.95	2.50	3.399 (9)	158
C69—H69*A*⋯O14^v^	0.98	2.44	3.323 (10)	150
C68—H68*A*⋯O10	0.98	2.55	3.214 (11)	125
C68—H68*A*⋯O9	0.98	2.66	3.224 (11)	117
C65—H65⋯O20^vi^	0.95	2.64	3.485 (8)	148
C61—H61⋯O13^vi^	0.95	2.49	3.429 (8)	172
C54—H54⋯O11^vi^	1.00	2.30	3.277 (8)	165
C46—H46⋯O25	0.95	2.32	3.211 (8)	155
C46—H46⋯O8^i^	0.95	2.56	3.054 (8)	113
C39—H39*A*⋯O27^i^	0.98	2.54	3.338 (9)	138
N38—H38⋯O18	0.86	1.87	2.550 (6)	135
N33—H33⋯O4	0.86	1.87	2.545 (7)	134
N37—H37⋯O17	0.83	1.89	2.582 (7)	139
N32—H32⋯O5	1.04	1.71	2.578 (6)	138

Symmetry codes: (i) -x+1, y, -z+1; (ii) -x+1, y, -z; (iii) x, y, z-1; (iv) -x+{\script{1\over 2}}, y-{\script{1\over 2}}, -z; (v) -x+{\script{1\over 2}}, y-{\script{1\over 2}}, -z+1; (vi) -x+{\script{1\over 2}}, y+{\script{1\over 2}}, -z+1.

**Table 2:** Experimental details

Crystal data	
Chemical formula	[Sm(NO_3_)_3_(C_30_H_28_N_2_O_4_)]
*M*_r_	816.92
Crystal system, space group	Monoclinic, *C*2
Temperature (K)	173
*a*, *b*, *c* (Å)	18.9105 (6), 15.7993 (5), 21.5738 (7)
β (°)	98.727 (1)
*V* (Å^3^)	6371.0 (4)
*Z*	8
Radiation type	Mo *K*α
μ (mm^−1^)	1.92
Crystal size (mm)	0.59 × 0.30 × 0.10

Data collection	
Diffractometer	Bruker APEXIII CCD
Absorption correction	Multi-scan
*T*_min_, *T*_max_	0.40, 0.83
No. of measured, independent and observed [*I* > 2σ(*I*)] reflections	40695, 15028, 12523
*R*_int_	0.042
(sin θ/λ)_max_ (Å^−1^)	0.732

Refinement	
*R*[*F* ^2^ > 2σ(*F* ^2^)], *wR*(*F* ^2^), *S*	0.033, 0.116, 0.83
No. of reflections	15028
No. of parameters	881
No. of restraints	49
H-atom treatment	H atoms treated by a mixture of independent and constrained refinement
Δρ_max_, Δρ_min_ (e Å^−3^)	0.84, −1.58
Absolute structure	Flack *x* determined using 4865 quotients [(*I* ^+^)−(*I* ^−^)]/[(*I* ^+^)+(*I* ^−^)] (Parsons *et al.*, 2013 ▸)
Absolute structure parameter	0.009 (9)

Computer programs: *APEX3* and *SAINT* (Bruker, 2017 ▸), *SHELXT2014/5* (Sheldrick, 2015*a* ▸), *SHELXL2016/6* (Sheldrick, 2015*b* ▸), *shelXle* (Hübschle *et al.*, 2011 ▸) and *SHELXTL* (Sheldrick, 2008 ▸).

## supplementary crystallographic information

### Crystal data

**Table:**

[Sm(NO_3_)_3_(C_30_H_28_N_2_O_4_)]	*F*(000) = 3272
*M**_r_* = 816.92	*D*_x_ = 1.703 Mg m^−^^3^
Monoclinic, *C*2	Mo *K*α radiation, λ = 0.71073 Å
*a* = 18.9105 (6) Å	Cell parameters from 859 reflections
*b* = 15.7993 (5) Å	θ = 1.7–28.6°
*c* = 21.5738 (7) Å	µ = 1.92 mm^−^^1^
β = 98.727 (1)°	*T* = 173 K
*V* = 6371.0 (4) Å^3^	Prism, yellow
*Z* = 8	0.59 × 0.30 × 0.10 mm

### Data collection

**Table:**

Bruker APEXIII CCD diffractometer	15028 independent reflections
Radiation source: fine-focus sealed tube	12523 reflections with *I* > 2σ(*I*)
Detector resolution: 7.3910 pixels mm^-1^	*R*_int_ = 0.042
φ and ω scans	θ_max_ = 31.3°, θ_min_ = 1.9°
Absorption correction: multi-scan	*h* = −27→27
*T*_min_ = 0.40, *T*_max_ = 0.83	*k* = −20→21
40695 measured reflections	*l* = −28→30

### Refinement

**Table:**

Refinement on *F*^2^	Hydrogen site location: mixed
Least-squares matrix: full	H atoms treated by a mixture of independent and constrained refinement
*R*[*F*^2^ > 2σ(*F*^2^)] = 0.033	*w* = 1/[σ^2^(*F*_o_^2^) + (0.1*P*)^2^ + 0.6514*P*] where *P* = (*F*_o_^2^ + 2*F*_c_^2^)/3
*wR*(*F*^2^) = 0.116	(Δ/σ)_max_ = 0.001
*S* = 0.83	Δρ_max_ = 0.84 e Å^−^^3^
15028 reflections	Δρ_min_ = −1.58 e Å^−^^3^
881 parameters	Absolute structure: Flack *x* determined using 4865 quotients [(*I*^+^)-(*I*^-^)]/[(*I*^+^)+(*I*^-^)] (Parsons *et al.*, 2013)
49 restraints	Absolute structure parameter: 0.009 (9)

### Special details

**Table:**

Geometry. All esds (except the esd in the dihedral angle between two l.s. planes) are estimated using the full covariance matrix. The cell esds are taken into account individually in the estimation of esds in distances, angles and torsion angles; correlations between esds in cell parameters are only used when they are defined by crystal symmetry. An approximate (isotropic) treatment of cell esds is used for estimating esds involving l.s. planes.

### Fractional atomic coordinates and isotropic or equivalent isotropic displacement parameters (Å^2^)

**Table:**

	*x*	*y*	*z*	*U*_iso_*/*U*_eq_	
Sm1	0.34946 (2)	0.59358 (2)	0.63113 (2)	0.03104 (10)	
Sm2	0.34751 (2)	0.39584 (2)	0.13107 (2)	0.03191 (11)	
O3	0.4131 (3)	0.4594 (4)	0.5968 (2)	0.0440 (13)	
O4	0.3665 (2)	0.5842 (4)	0.52494 (19)	0.0336 (10)	
O5	0.3218 (3)	0.7237 (3)	0.5804 (2)	0.0342 (11)	
O6	0.3492 (3)	0.7271 (4)	0.7027 (2)	0.0398 (12)	
H32	0.312210	0.749766	0.502781	0.05 (2)*	
O7	0.4735 (3)	0.6447 (5)	0.6256 (3)	0.065 (2)	
O9	0.4581 (3)	0.5828 (6)	0.7107 (2)	0.0519 (16)	
O8	0.5637 (3)	0.6337 (7)	0.7015 (3)	0.081 (3)	
O10	0.3289 (3)	0.5319 (4)	0.7402 (2)	0.0497 (14)	
O11	0.2958 (3)	0.4561 (4)	0.6585 (2)	0.0401 (12)	
O12	0.2771 (3)	0.4098 (5)	0.7490 (2)	0.0501 (16)	
O13	0.2293 (3)	0.5689 (4)	0.5608 (2)	0.0416 (13)	
O14	0.2255 (3)	0.6244 (4)	0.6515 (2)	0.0509 (15)	
O15	0.1278 (3)	0.6161 (6)	0.5828 (4)	0.081 (3)	
O16	0.4178 (3)	0.5280 (4)	0.0995 (2)	0.0423 (13)	
O17	0.3733 (2)	0.4025 (4)	0.02689 (18)	0.0361 (11)	
O18	0.3128 (3)	0.2709 (3)	0.0773 (2)	0.0392 (11)	
O19	0.3455 (3)	0.2594 (4)	0.1986 (2)	0.0449 (13)	
O20	0.2196 (3)	0.3731 (4)	0.1473 (2)	0.0473 (13)	
O21	0.2315 (3)	0.4286 (4)	0.0572 (2)	0.0438 (14)	
O22	0.1272 (3)	0.3899 (7)	0.0772 (3)	0.082 (2)	
O23	0.3243 (3)	0.4607 (3)	0.2386 (2)	0.0399 (12)	
H37	0.336816	0.342349	−0.046833	0.06 (2)*	
O24	0.2953 (3)	0.5368 (4)	0.1565 (2)	0.0407 (13)	
O25	0.2780 (2)	0.5849 (4)	0.24702 (19)	0.0387 (11)	
O26	0.4537 (3)	0.4042 (5)	0.2154 (2)	0.0456 (13)	
O27	0.5601 (3)	0.3585 (5)	0.2082 (2)	0.0656 (19)	
O28	0.4709 (3)	0.3425 (6)	0.1312 (3)	0.074 (2)	
N29	0.1918 (3)	0.6040 (6)	0.5969 (3)	0.0446 (17)	
N30	0.3004 (3)	0.4638 (5)	0.7169 (3)	0.0369 (15)	
N31	0.5005 (3)	0.6206 (7)	0.6799 (3)	0.059 (3)	
N32	0.3003 (2)	0.8029 (3)	0.4747 (2)	0.0295 (10)	
N33	0.3249 (3)	0.6451 (4)	0.4163 (2)	0.0274 (10)	
H33	0.330349	0.652121	0.456298	0.033*	
N34	0.1893 (3)	0.3978 (6)	0.0936 (3)	0.0458 (15)	
N35	0.2982 (3)	0.5279 (4)	0.2156 (2)	0.0334 (14)	
N36	0.4974 (3)	0.3681 (5)	0.1853 (3)	0.0427 (18)	
N37	0.3445 (3)	0.3371 (4)	−0.0837 (2)	0.0290 (11)	
N38	0.2753 (2)	0.2045 (3)	−0.0304 (2)	0.0283 (10)	
H38	0.281783	0.249361	−0.007628	0.034*	
C39	0.4367 (4)	0.3910 (7)	0.6385 (3)	0.051 (2)	
H39A	0.445994	0.412274	0.681699	0.077*	
H39B	0.480660	0.366718	0.627254	0.077*	
H39C	0.399464	0.347401	0.635258	0.077*	
C40	0.4033 (4)	0.4420 (5)	0.5336 (3)	0.0346 (17)	
C41	0.4114 (3)	0.3647 (4)	0.5056 (3)	0.0370 (17)	
H41	0.425050	0.316532	0.530957	0.044*	
C42	0.3999 (4)	0.3567 (5)	0.4409 (3)	0.0450 (18)	
H42	0.404084	0.303086	0.421899	0.054*	
C43	0.3821 (4)	0.4278 (5)	0.4040 (3)	0.0390 (17)	
H43	0.377368	0.423280	0.359672	0.047*	
C44	0.3710 (3)	0.5060 (5)	0.4313 (3)	0.0306 (14)	
C45	0.3808 (3)	0.5142 (5)	0.4972 (3)	0.0261 (13)	
C46	0.3426 (3)	0.5731 (5)	0.3933 (3)	0.0287 (15)	
H46	0.336029	0.566135	0.349023	0.034*	
C47	0.2838 (3)	0.7123 (4)	0.3798 (2)	0.0294 (11)	
H47	0.296223	0.710575	0.336410	0.035*	
C48	0.2038 (3)	0.6939 (4)	0.3748 (3)	0.0314 (13)	
C49	0.1730 (3)	0.6685 (5)	0.4268 (3)	0.0353 (14)	
H49	0.201824	0.663385	0.466731	0.042*	
C50	0.1002 (4)	0.6509 (5)	0.4202 (4)	0.0455 (18)	
H50	0.079376	0.633347	0.455476	0.055*	
C51	0.0585 (4)	0.6587 (5)	0.3627 (4)	0.051 (2)	
H51	0.008972	0.645444	0.358318	0.062*	
C53	0.1603 (4)	0.7029 (5)	0.3165 (3)	0.0361 (15)	
H53	0.180623	0.720595	0.280987	0.043*	
C52	0.0877 (4)	0.6858 (6)	0.3107 (4)	0.0477 (18)	
H52	0.058045	0.692689	0.271375	0.057*	
C54	0.3039 (3)	0.8011 (4)	0.4069 (2)	0.0307 (11)	
H54	0.266389	0.840983	0.386531	0.037*	
C55	0.37537 (19)	0.8350 (3)	0.3938 (3)	0.0392 (13)	
C56	0.3759 (2)	0.9062 (4)	0.3560 (3)	0.069 (2)	
H56	0.332134	0.931450	0.337664	0.083*	
C57	0.4406 (3)	0.9405 (4)	0.3451 (3)	0.079 (3)	
H57	0.441010	0.989164	0.319282	0.095*	
C58	0.5047 (2)	0.9036 (5)	0.3720 (4)	0.073 (2)	
H58	0.548925	0.927044	0.364509	0.087*	
C59	0.50415 (19)	0.8324 (5)	0.4098 (4)	0.118 (4)	
H59	0.547964	0.807212	0.428118	0.141*	
C60	0.4395 (2)	0.7981 (4)	0.4207 (3)	0.106 (3)	
H60	0.439088	0.749498	0.446501	0.128*	
C61	0.2952 (3)	0.8720 (4)	0.5059 (3)	0.0283 (12)	
H61	0.285957	0.923758	0.483751	0.034*	
C62	0.3030 (3)	0.8722 (5)	0.5719 (3)	0.0312 (15)	
C63	0.3003 (4)	0.9513 (5)	0.6042 (4)	0.0373 (16)	
H63	0.290380	1.002088	0.580941	0.045*	
C64	0.3119 (4)	0.9538 (6)	0.6681 (4)	0.0469 (19)	
H64	0.309507	1.006212	0.689194	0.056*	
C65	0.3274 (4)	0.8795 (5)	0.7026 (3)	0.0397 (17)	
H65	0.334973	0.882498	0.747141	0.048*	
C66	0.3318 (4)	0.8029 (5)	0.6744 (3)	0.0354 (15)	
C67	0.3177 (3)	0.7977 (5)	0.6067 (3)	0.0281 (14)	
C68	0.3680 (4)	0.7271 (6)	0.7700 (3)	0.0499 (19)	
H68A	0.384219	0.670427	0.784231	0.075*	
H68B	0.326080	0.742748	0.789153	0.075*	
H68C	0.406501	0.768006	0.782367	0.075*	
C69	0.3627 (5)	0.2536 (6)	0.2657 (3)	0.057 (2)	
H69A	0.322245	0.228728	0.282772	0.085*	
H69B	0.372451	0.310348	0.283318	0.085*	
H69C	0.405073	0.217886	0.276765	0.085*	
C70	0.3310 (4)	0.1831 (5)	0.1664 (3)	0.0342 (15)	
C71	0.3305 (4)	0.1039 (6)	0.1932 (3)	0.0419 (17)	
H71	0.338944	0.097875	0.237482	0.050*	
C72	0.3176 (4)	0.0323 (5)	0.1545 (4)	0.0423 (17)	
H72	0.318484	−0.022487	0.172839	0.051*	
C73	0.3036 (4)	0.0408 (5)	0.0901 (3)	0.0387 (16)	
H73	0.296486	−0.008063	0.064178	0.046*	
C74	0.3000 (3)	0.1208 (5)	0.0631 (3)	0.0323 (16)	
C75	0.3144 (4)	0.1955 (5)	0.1012 (3)	0.0326 (15)	
C76	0.2819 (3)	0.1313 (4)	−0.0040 (3)	0.0307 (13)	
H76	0.274596	0.082215	−0.029571	0.037*	
C77	0.2589 (3)	0.2203 (3)	−0.0980 (2)	0.0289 (10)	
H77	0.251341	0.164019	−0.119107	0.035*	
C78	0.1892 (3)	0.2715 (4)	−0.1156 (3)	0.0320 (13)	
C79	0.1580 (5)	0.2715 (5)	−0.1764 (4)	0.0464 (19)	
H79	0.181222	0.243622	−0.206714	0.056*	
C80	0.0928 (5)	0.3114 (6)	−0.1954 (4)	0.062 (2)	
H80	0.072411	0.311722	−0.238458	0.074*	
C81	0.0583 (4)	0.3498 (6)	−0.1523 (5)	0.064 (3)	
H81	0.012808	0.375385	−0.164474	0.077*	
C82	0.0911 (4)	0.3511 (6)	−0.0897 (5)	0.057 (2)	
H82	0.059 (4)	0.370 (6)	−0.064 (4)	0.06 (2)*	
C83	0.1553 (4)	0.3129 (5)	−0.0719 (4)	0.0423 (16)	
H83	0.176979	0.314561	−0.029180	0.051*	
C84	0.3251 (3)	0.2616 (4)	−0.1208 (2)	0.0329 (11)	
H84	0.311382	0.278765	−0.165589	0.039*	
C86	0.3769 (3)	0.1272 (4)	−0.1558 (3)	0.0393 (14)	
H86	0.333439	0.119803	−0.183756	0.047*	
C85	0.3858 (3)	0.1973 (4)	−0.1169 (3)	0.0322 (11)	
C87	0.4309 (4)	0.0681 (6)	−0.1540 (4)	0.0556 (19)	
H87	0.422910	0.019203	−0.179743	0.067*	
C89	0.5036 (4)	0.1445 (7)	−0.0774 (5)	0.088 (4)	
H89	0.546499	0.149087	−0.048387	0.106*	
C88	0.4945 (4)	0.0776 (6)	−0.1170 (5)	0.063 (2)	
H88	0.532161	0.038266	−0.118688	0.076*	
C90	0.4500 (4)	0.2080 (5)	−0.0785 (4)	0.0558 (18)	
H90	0.458592	0.257054	−0.052928	0.067*	
C91	0.3504 (3)	0.4121 (5)	−0.1058 (3)	0.0297 (14)	
H91	0.341392	0.419984	−0.149996	0.036*	
C92	0.3701 (3)	0.4843 (5)	−0.0666 (3)	0.0269 (13)	
C93	0.3750 (4)	0.5661 (5)	−0.0934 (4)	0.0389 (17)	
H93	0.364489	0.573123	−0.137529	0.047*	
C94	0.3946 (4)	0.6338 (5)	−0.0564 (4)	0.0402 (16)	
H94	0.397898	0.687958	−0.074857	0.048*	
C95	0.4104 (4)	0.6247 (5)	0.0098 (3)	0.0403 (19)	
H95	0.424791	0.672383	0.035450	0.048*	
C96	0.4046 (3)	0.5466 (5)	0.0363 (3)	0.0302 (15)	
C97	0.3825 (3)	0.4752 (5)	−0.0004 (3)	0.0324 (15)	
C98	0.4469 (4)	0.5942 (7)	0.1414 (3)	0.0467 (18)	
H98A	0.456507	0.572119	0.184323	0.070*	
H98B	0.412560	0.640895	0.139544	0.070*	
H98C	0.491636	0.614798	0.129001	0.070*	

### Atomic displacement parameters (Å^2^)

**Table:**

	*U*^11^	*U*^22^	*U*^33^	*U*^12^	*U*^13^	*U*^23^
Sm1	0.0435 (2)	0.0290 (2)	0.02008 (18)	0.00589 (17)	0.00313 (14)	−0.00049 (14)
Sm2	0.0474 (2)	0.0301 (2)	0.01812 (17)	−0.00325 (17)	0.00465 (15)	−0.00037 (14)
O3	0.064 (3)	0.034 (3)	0.036 (3)	0.019 (3)	0.013 (2)	0.010 (2)
O4	0.050 (2)	0.028 (3)	0.0227 (19)	0.010 (2)	0.0059 (17)	0.001 (2)
O5	0.054 (3)	0.024 (2)	0.024 (2)	0.005 (2)	0.0048 (18)	−0.0034 (19)
O6	0.059 (3)	0.033 (3)	0.028 (2)	0.002 (3)	0.005 (2)	−0.004 (2)
O7	0.054 (3)	0.107 (6)	0.033 (3)	−0.017 (4)	0.004 (2)	0.002 (3)
O9	0.048 (3)	0.071 (5)	0.034 (2)	0.010 (3)	−0.001 (2)	0.004 (3)
O8	0.042 (3)	0.157 (8)	0.044 (3)	0.005 (4)	0.000 (2)	−0.007 (4)
O10	0.072 (3)	0.044 (3)	0.031 (3)	−0.018 (3)	0.001 (2)	−0.011 (3)
O11	0.048 (3)	0.036 (3)	0.036 (3)	−0.004 (2)	0.005 (2)	−0.007 (2)
O12	0.052 (3)	0.052 (4)	0.043 (3)	−0.018 (3)	−0.004 (2)	0.011 (3)
O13	0.050 (3)	0.042 (3)	0.032 (2)	0.004 (2)	0.003 (2)	0.004 (2)
O14	0.061 (3)	0.056 (4)	0.038 (3)	0.012 (3)	0.016 (2)	−0.005 (3)
O15	0.052 (3)	0.096 (6)	0.093 (5)	0.019 (4)	0.009 (3)	0.024 (5)
O16	0.057 (3)	0.050 (4)	0.021 (2)	−0.014 (3)	0.007 (2)	−0.009 (2)
O17	0.060 (3)	0.029 (3)	0.0190 (19)	−0.009 (3)	0.0066 (18)	−0.001 (2)
O18	0.064 (3)	0.029 (3)	0.026 (2)	−0.005 (2)	0.007 (2)	0.004 (2)
O19	0.076 (3)	0.038 (3)	0.020 (2)	−0.007 (3)	0.004 (2)	0.007 (2)
O20	0.053 (3)	0.049 (3)	0.042 (3)	−0.008 (3)	0.012 (2)	0.001 (2)
O21	0.054 (3)	0.049 (3)	0.026 (2)	0.003 (3)	−0.001 (2)	0.004 (2)
O22	0.043 (3)	0.126 (7)	0.074 (4)	−0.004 (4)	0.001 (3)	−0.025 (5)
O23	0.061 (3)	0.033 (3)	0.024 (2)	0.005 (3)	0.001 (2)	−0.001 (2)
O24	0.068 (3)	0.036 (3)	0.019 (2)	−0.001 (3)	0.011 (2)	0.004 (2)
O25	0.049 (2)	0.040 (3)	0.027 (2)	−0.001 (3)	0.0064 (18)	−0.009 (2)
O26	0.053 (3)	0.057 (4)	0.028 (2)	−0.002 (3)	0.010 (2)	−0.006 (3)
O27	0.053 (3)	0.101 (6)	0.044 (3)	0.007 (3)	0.009 (2)	−0.003 (3)
O28	0.063 (4)	0.119 (7)	0.038 (3)	0.014 (4)	0.000 (3)	−0.035 (4)
N29	0.041 (3)	0.046 (4)	0.047 (4)	0.008 (3)	0.006 (3)	0.010 (3)
N30	0.046 (3)	0.037 (4)	0.028 (3)	0.000 (3)	0.005 (2)	0.006 (3)
N31	0.039 (3)	0.099 (8)	0.037 (3)	0.007 (4)	0.002 (3)	−0.011 (4)
N32	0.030 (2)	0.025 (3)	0.032 (2)	0.002 (2)	0.0013 (18)	0.0037 (18)
N33	0.032 (2)	0.027 (3)	0.024 (2)	0.003 (2)	0.0046 (18)	0.0007 (19)
N34	0.048 (3)	0.042 (4)	0.047 (4)	−0.001 (3)	0.007 (3)	−0.016 (3)
N35	0.034 (3)	0.034 (4)	0.029 (3)	−0.004 (3)	−0.002 (2)	0.001 (3)
N36	0.046 (3)	0.055 (5)	0.028 (3)	0.000 (3)	0.005 (2)	−0.004 (3)
N37	0.034 (2)	0.034 (3)	0.018 (2)	0.001 (2)	0.0028 (18)	−0.0017 (19)
N38	0.033 (2)	0.028 (2)	0.024 (2)	0.001 (2)	0.0035 (17)	−0.0024 (18)
C39	0.071 (5)	0.048 (5)	0.037 (4)	0.022 (5)	0.015 (3)	0.012 (4)
C40	0.045 (4)	0.031 (4)	0.029 (3)	0.003 (3)	0.010 (3)	0.004 (3)
C41	0.042 (3)	0.019 (4)	0.052 (4)	0.009 (3)	0.014 (3)	0.006 (3)
C42	0.059 (4)	0.037 (4)	0.039 (4)	0.005 (4)	0.008 (3)	−0.017 (3)
C43	0.050 (4)	0.036 (4)	0.029 (3)	0.007 (3)	0.001 (3)	−0.007 (3)
C44	0.029 (3)	0.035 (4)	0.027 (3)	0.002 (3)	0.002 (2)	−0.006 (3)
C45	0.026 (3)	0.031 (4)	0.021 (3)	0.003 (3)	0.000 (2)	−0.001 (3)
C46	0.027 (3)	0.037 (4)	0.022 (3)	−0.001 (3)	0.001 (2)	−0.005 (3)
C47	0.038 (3)	0.030 (3)	0.021 (2)	0.002 (2)	0.005 (2)	0.004 (2)
C48	0.031 (3)	0.024 (3)	0.036 (3)	0.004 (2)	−0.004 (2)	0.003 (2)
C49	0.034 (3)	0.032 (3)	0.038 (3)	−0.006 (3)	−0.002 (2)	0.003 (3)
C50	0.039 (3)	0.036 (4)	0.062 (4)	−0.004 (3)	0.010 (3)	0.011 (3)
C51	0.029 (3)	0.037 (4)	0.083 (6)	−0.008 (3)	−0.008 (3)	−0.005 (4)
C53	0.042 (3)	0.033 (4)	0.030 (3)	0.007 (3)	−0.004 (2)	−0.001 (3)
C52	0.040 (3)	0.041 (4)	0.054 (4)	−0.005 (3)	−0.018 (3)	0.000 (4)
C54	0.033 (2)	0.030 (3)	0.029 (2)	−0.002 (2)	0.005 (2)	0.003 (2)
C55	0.027 (2)	0.036 (3)	0.055 (3)	−0.002 (3)	0.009 (2)	0.002 (3)
C56	0.048 (4)	0.084 (6)	0.070 (5)	−0.020 (4)	−0.005 (3)	0.038 (4)
C57	0.050 (4)	0.092 (6)	0.093 (6)	−0.036 (4)	0.003 (4)	0.032 (5)
C58	0.040 (3)	0.076 (6)	0.106 (6)	−0.009 (4)	0.024 (4)	0.010 (5)
C59	0.044 (4)	0.087 (7)	0.223 (9)	−0.007 (5)	0.020 (6)	0.060 (7)
C60	0.039 (4)	0.076 (6)	0.202 (8)	−0.005 (4)	0.012 (5)	0.065 (6)
C61	0.026 (2)	0.021 (3)	0.038 (3)	0.006 (2)	0.003 (2)	0.002 (2)
C62	0.023 (3)	0.031 (4)	0.039 (3)	0.006 (3)	0.004 (2)	−0.001 (3)
C63	0.037 (3)	0.022 (3)	0.055 (4)	0.004 (3)	0.014 (3)	−0.009 (3)
C64	0.047 (4)	0.035 (4)	0.061 (5)	0.001 (3)	0.016 (3)	−0.020 (3)
C65	0.042 (3)	0.041 (4)	0.040 (3)	−0.005 (3)	0.017 (3)	−0.014 (3)
C66	0.042 (3)	0.041 (4)	0.025 (3)	−0.001 (3)	0.010 (3)	−0.009 (3)
C67	0.028 (3)	0.023 (3)	0.034 (3)	0.004 (3)	0.005 (2)	−0.009 (3)
C68	0.076 (5)	0.046 (4)	0.026 (3)	−0.004 (4)	0.001 (3)	−0.008 (3)
C69	0.093 (6)	0.050 (5)	0.025 (3)	−0.001 (5)	0.001 (4)	0.011 (3)
C70	0.039 (3)	0.028 (4)	0.035 (3)	−0.006 (3)	0.006 (3)	0.006 (3)
C71	0.043 (3)	0.039 (4)	0.043 (4)	−0.004 (3)	0.002 (3)	0.017 (3)
C72	0.039 (3)	0.032 (4)	0.054 (4)	−0.001 (3)	0.001 (3)	0.016 (3)
C73	0.039 (3)	0.033 (4)	0.043 (4)	−0.007 (3)	0.006 (3)	0.006 (3)
C74	0.030 (3)	0.027 (4)	0.040 (3)	−0.004 (3)	0.009 (3)	0.001 (3)
C75	0.038 (3)	0.033 (4)	0.029 (3)	0.001 (3)	0.011 (2)	0.008 (3)
C76	0.029 (2)	0.025 (3)	0.040 (3)	−0.003 (2)	0.009 (2)	−0.001 (2)
C77	0.034 (3)	0.026 (3)	0.025 (2)	0.001 (2)	−0.0019 (19)	−0.0051 (19)
C78	0.035 (3)	0.021 (3)	0.038 (3)	0.000 (3)	−0.003 (2)	−0.006 (2)
C79	0.053 (4)	0.034 (4)	0.048 (4)	0.005 (3)	−0.005 (3)	−0.002 (3)
C80	0.066 (5)	0.038 (5)	0.069 (5)	0.002 (4)	−0.029 (4)	−0.002 (4)
C81	0.046 (4)	0.031 (4)	0.104 (7)	0.010 (3)	−0.023 (4)	−0.011 (4)
C82	0.048 (4)	0.033 (4)	0.089 (6)	0.001 (3)	0.011 (4)	−0.014 (4)
C83	0.045 (3)	0.029 (3)	0.053 (4)	−0.004 (3)	0.009 (3)	−0.011 (3)
C84	0.045 (3)	0.033 (3)	0.021 (2)	0.000 (3)	0.006 (2)	−0.003 (2)
C86	0.040 (3)	0.033 (3)	0.043 (3)	0.006 (3)	−0.001 (2)	−0.013 (3)
C85	0.036 (3)	0.032 (3)	0.031 (3)	−0.001 (2)	0.013 (2)	0.000 (2)
C87	0.061 (4)	0.043 (4)	0.064 (5)	0.011 (4)	0.013 (4)	−0.014 (4)
C89	0.022 (3)	0.095 (8)	0.143 (9)	0.004 (4)	−0.004 (4)	−0.027 (7)
C88	0.048 (4)	0.040 (4)	0.105 (7)	0.012 (4)	0.023 (4)	−0.005 (5)
C90	0.041 (3)	0.047 (4)	0.080 (5)	−0.006 (3)	0.010 (3)	−0.011 (4)
C91	0.033 (3)	0.031 (4)	0.025 (3)	0.000 (3)	0.005 (2)	0.000 (3)
C92	0.026 (3)	0.029 (3)	0.026 (3)	−0.003 (3)	0.004 (2)	0.000 (3)
C93	0.042 (4)	0.031 (4)	0.043 (4)	−0.001 (3)	0.003 (3)	0.006 (3)
C94	0.044 (4)	0.023 (3)	0.054 (4)	−0.005 (3)	0.008 (3)	−0.003 (3)
C95	0.042 (4)	0.037 (5)	0.043 (4)	−0.010 (3)	0.012 (3)	−0.009 (3)
C96	0.028 (3)	0.030 (4)	0.033 (3)	−0.005 (3)	0.006 (3)	−0.004 (3)
C97	0.038 (3)	0.024 (4)	0.038 (4)	−0.006 (3)	0.015 (3)	−0.006 (3)
C98	0.050 (3)	0.045 (4)	0.046 (4)	−0.013 (4)	0.009 (3)	−0.024 (4)

### Geometric parameters (Å, º)

**Table:**

Sm1—O5	2.350 (5)	C50—H50	0.9500
Sm1—O4	2.366 (4)	C51—C52	1.392 (11)
Sm1—O9	2.476 (5)	C51—H51	0.9500
Sm1—O14	2.498 (5)	C53—C52	1.386 (10)
Sm1—O7	2.500 (6)	C53—H53	0.9500
Sm1—O11	2.505 (6)	C52—H52	0.9500
Sm1—O13	2.563 (5)	C54—C55	1.520 (6)
Sm1—O3	2.601 (5)	C54—H54	1.0000
Sm1—O6	2.614 (5)	C55—C56	1.3900
Sm1—O10	2.630 (5)	C55—C60	1.3900
Sm1—N31	2.922 (6)	C56—C57	1.3900
Sm1—N29	2.965 (6)	C56—H56	0.9500
Sm2—O18	2.333 (5)	C57—C58	1.3900
Sm2—O17	2.373 (4)	C57—H57	0.9500
Sm2—O28	2.481 (6)	C58—C59	1.3900
Sm2—O26	2.500 (5)	C58—H58	0.9500
Sm2—O20	2.522 (5)	C59—C60	1.3900
Sm2—O24	2.530 (6)	C59—H59	0.9500
Sm2—O21	2.561 (5)	C60—H60	0.9500
Sm2—O19	2.606 (5)	C61—C62	1.409 (9)
Sm2—O16	2.621 (6)	C61—H61	0.9500
Sm2—O23	2.634 (4)	C62—C67	1.402 (10)
Sm2—N36	2.930 (6)	C62—C63	1.435 (10)
Sm2—N34	2.978 (6)	C63—C64	1.363 (11)
O3—C40	1.375 (8)	C63—H63	0.9500
O3—C39	1.433 (10)	C64—C65	1.397 (12)
O4—C45	1.304 (8)	C64—H64	0.9500
O5—C67	1.306 (8)	C65—C66	1.363 (11)
O6—C66	1.362 (10)	C65—H65	0.9500
O6—C68	1.442 (7)	C66—C67	1.447 (9)
O7—N31	1.263 (9)	C68—H68A	0.9800
O9—N31	1.267 (10)	C68—H68B	0.9800
O8—N31	1.232 (8)	C68—H68C	0.9800
O10—N30	1.272 (9)	C69—H69A	0.9800
O11—N30	1.255 (7)	C69—H69B	0.9800
O12—N30	1.223 (8)	C69—H69C	0.9800
O13—N29	1.259 (8)	C70—C71	1.378 (11)
O14—N29	1.291 (9)	C70—C75	1.408 (10)
O15—N29	1.219 (9)	C71—C72	1.406 (12)
O16—C96	1.380 (8)	C71—H71	0.9500
O16—C98	1.437 (10)	C72—C73	1.382 (11)
O17—C97	1.315 (9)	C72—H72	0.9500
O18—C75	1.295 (9)	C73—C74	1.389 (11)
O19—C70	1.397 (9)	C73—H73	0.9500
O19—C69	1.438 (7)	C74—C75	1.439 (11)
O20—N34	1.273 (9)	C74—C76	1.445 (9)
O21—N34	1.296 (9)	C76—H76	0.9500
O22—N34	1.181 (8)	C77—C78	1.544 (8)
O23—N35	1.241 (8)	C77—C84	1.555 (7)
O24—N35	1.278 (7)	C77—H77	1.0000
O25—N35	1.222 (8)	C78—C79	1.353 (10)
O26—N36	1.260 (8)	C78—C83	1.383 (9)
O27—N36	1.223 (8)	C79—C80	1.391 (12)
O28—N36	1.265 (8)	C79—H79	0.9500
N32—C61	1.292 (8)	C80—C81	1.357 (12)
N32—C54	1.475 (6)	C80—H80	0.9500
N32—H32	1.0395	C81—C82	1.399 (14)
N33—C46	1.305 (9)	C81—H81	0.9500
N33—C47	1.473 (7)	C82—C83	1.358 (11)
N33—H33	0.8600	C82—H82	0.92 (8)
N37—C91	1.290 (9)	C83—H83	0.9500
N37—C84	1.451 (8)	C84—C85	1.526 (8)
N37—H37	0.8345	C84—H84	1.0000
N38—C76	1.287 (8)	C86—C87	1.380 (10)
N38—C77	1.466 (6)	C86—C85	1.383 (8)
N38—H38	0.8600	C86—H86	0.9500
C39—H39A	0.9800	C85—C90	1.374 (9)
C39—H39B	0.9800	C87—C88	1.349 (12)
C39—H39C	0.9800	C87—H87	0.9500
C40—C41	1.381 (10)	C89—C88	1.352 (13)
C40—C45	1.414 (10)	C89—C90	1.424 (12)
C41—C42	1.386 (9)	C89—H89	0.9500
C41—H41	0.9500	C88—H88	0.9500
C42—C43	1.387 (12)	C90—H90	0.9500
C42—H42	0.9500	C91—C92	1.436 (10)
C43—C44	1.397 (11)	C91—H91	0.9500
C43—H43	0.9500	C92—C97	1.420 (9)
C44—C46	1.397 (11)	C92—C93	1.424 (11)
C44—C45	1.413 (8)	C93—C94	1.352 (11)
C46—H46	0.9500	C93—H93	0.9500
C47—C48	1.527 (8)	C94—C95	1.421 (10)
C47—C54	1.545 (8)	C94—H94	0.9500
C47—H47	1.0000	C95—C96	1.371 (11)
C48—C49	1.399 (9)	C95—H95	0.9500
C48—C53	1.402 (9)	C96—C97	1.404 (9)
C49—C50	1.391 (9)	C98—H98A	0.9800
C49—H49	0.9500	C98—H98B	0.9800
C50—C51	1.370 (12)	C98—H98C	0.9800
			
O5—Sm1—O4	69.96 (17)	O3—C39—H39B	109.5
O5—Sm1—O9	119.1 (2)	H39A—C39—H39B	109.5
O4—Sm1—O9	116.53 (16)	O3—C39—H39C	109.5
O5—Sm1—O14	76.29 (18)	H39A—C39—H39C	109.5
O4—Sm1—O14	116.86 (16)	H39B—C39—H39C	109.5
O9—Sm1—O14	126.43 (17)	O3—C40—C41	127.1 (7)
O5—Sm1—O7	80.7 (2)	O3—C40—C45	111.9 (6)
O4—Sm1—O7	72.98 (17)	C41—C40—C45	121.0 (6)
O9—Sm1—O7	50.9 (2)	C40—C41—C42	120.7 (7)
O14—Sm1—O7	149.1 (3)	C40—C41—H41	119.7
O5—Sm1—O11	143.01 (17)	C42—C41—H41	119.7
O4—Sm1—O11	107.06 (18)	C41—C42—C43	119.4 (7)
O9—Sm1—O11	95.7 (2)	C41—C42—H42	120.3
O14—Sm1—O11	72.73 (19)	C43—C42—H42	120.3
O7—Sm1—O11	135.0 (2)	C42—C43—C44	120.9 (6)
O5—Sm1—O13	75.13 (18)	C42—C43—H43	119.6
O4—Sm1—O13	69.89 (14)	C44—C43—H43	119.6
O9—Sm1—O13	165.4 (2)	C46—C44—C43	119.5 (6)
O14—Sm1—O13	50.38 (17)	C46—C44—C45	120.1 (6)
O7—Sm1—O13	140.77 (17)	C43—C44—C45	120.0 (7)
O11—Sm1—O13	69.70 (17)	O4—C45—C44	122.4 (6)
O5—Sm1—O3	131.33 (15)	O4—C45—C40	119.7 (5)
O4—Sm1—O3	62.37 (17)	C44—C45—C40	117.8 (6)
O9—Sm1—O3	76.7 (2)	N33—C46—C44	122.4 (6)
O14—Sm1—O3	134.1 (2)	N33—C46—H46	118.8
O7—Sm1—O3	76.8 (2)	C44—C46—H46	118.8
O11—Sm1—O3	65.28 (18)	N33—C47—C48	109.7 (5)
O13—Sm1—O3	96.56 (19)	N33—C47—C54	111.9 (4)
O5—Sm1—O6	63.17 (16)	C48—C47—C54	112.6 (5)
O4—Sm1—O6	129.40 (18)	N33—C47—H47	107.5
O9—Sm1—O6	73.8 (2)	C48—C47—H47	107.5
O14—Sm1—O6	69.72 (18)	C54—C47—H47	107.5
O7—Sm1—O6	81.6 (2)	C49—C48—C53	119.3 (6)
O11—Sm1—O6	121.57 (16)	C49—C48—C47	121.8 (5)
O13—Sm1—O6	113.06 (17)	C53—C48—C47	118.9 (6)
O3—Sm1—O6	150.26 (17)	C50—C49—C48	120.1 (7)
O5—Sm1—O10	133.49 (17)	C50—C49—H49	120.0
O4—Sm1—O10	154.62 (18)	C48—C49—H49	120.0
O9—Sm1—O10	64.75 (18)	C51—C50—C49	120.0 (7)
O14—Sm1—O10	69.20 (18)	C51—C50—H50	120.0
O7—Sm1—O10	115.43 (18)	C49—C50—H50	120.0
O11—Sm1—O10	49.29 (17)	C50—C51—C52	120.8 (7)
O13—Sm1—O10	103.59 (16)	C50—C51—H51	119.6
O3—Sm1—O10	95.18 (18)	C52—C51—H51	119.6
O6—Sm1—O10	75.95 (17)	C52—C53—C48	120.0 (7)
O5—Sm1—N31	100.1 (2)	C52—C53—H53	120.0
O4—Sm1—N31	95.06 (16)	C48—C53—H53	120.0
O9—Sm1—N31	25.5 (2)	C53—C52—C51	119.8 (7)
O14—Sm1—N31	143.2 (2)	C53—C52—H52	120.1
O7—Sm1—N31	25.4 (2)	C51—C52—H52	120.1
O11—Sm1—N31	116.8 (2)	N32—C54—C55	110.7 (4)
O13—Sm1—N31	164.95 (16)	N32—C54—C47	110.3 (4)
O3—Sm1—N31	75.6 (2)	C55—C54—C47	115.2 (4)
O6—Sm1—N31	76.0 (2)	N32—C54—H54	106.7
O10—Sm1—N31	90.08 (19)	C55—C54—H54	106.7
O5—Sm1—N29	72.0 (2)	C47—C54—H54	106.7
O4—Sm1—N29	92.41 (17)	C56—C55—C60	120.0
O9—Sm1—N29	150.90 (17)	C56—C55—C54	118.8 (4)
O14—Sm1—N29	25.56 (18)	C60—C55—C54	121.2 (4)
O7—Sm1—N29	152.3 (2)	C55—C56—C57	120.0
O11—Sm1—N29	71.3 (2)	C55—C56—H56	120.0
O13—Sm1—N29	24.99 (18)	C57—C56—H56	120.0
O3—Sm1—N29	117.7 (2)	C58—C57—C56	120.0
O6—Sm1—N29	90.6 (2)	C58—C57—H57	120.0
O10—Sm1—N29	87.92 (18)	C56—C57—H57	120.0
N31—Sm1—N29	166.6 (3)	C59—C58—C57	120.0
O18—Sm2—O17	69.48 (17)	C59—C58—H58	120.0
O18—Sm2—O28	84.7 (2)	C57—C58—H58	120.0
O17—Sm2—O28	71.80 (19)	C60—C59—C58	120.0
O18—Sm2—O26	122.7 (2)	C60—C59—H59	120.0
O17—Sm2—O26	115.40 (15)	C58—C59—H59	120.0
O28—Sm2—O26	50.61 (17)	C59—C60—C55	120.0
O18—Sm2—O20	75.22 (19)	C59—C60—H60	120.0
O17—Sm2—O20	118.48 (16)	C55—C60—H60	120.0
O28—Sm2—O20	150.9 (3)	N32—C61—C62	121.5 (6)
O26—Sm2—O20	126.03 (15)	N32—C61—H61	119.2
O18—Sm2—O24	139.72 (18)	C62—C61—H61	119.2
O17—Sm2—O24	108.00 (18)	C67—C62—C61	121.4 (6)
O28—Sm2—O24	134.2 (2)	C67—C62—C63	119.3 (6)
O26—Sm2—O24	95.3 (2)	C61—C62—C63	119.2 (7)
O20—Sm2—O24	71.40 (19)	C64—C63—C62	120.3 (8)
O18—Sm2—O21	73.25 (19)	C64—C63—H63	119.9
O17—Sm2—O21	71.42 (15)	C62—C63—H63	119.9
O28—Sm2—O21	141.83 (17)	C63—C64—C65	120.3 (7)
O26—Sm2—O21	163.7 (2)	C63—C64—H64	119.9
O20—Sm2—O21	50.65 (17)	C65—C64—H64	119.9
O24—Sm2—O21	68.35 (18)	C66—C65—C64	122.0 (7)
O18—Sm2—O19	63.28 (17)	C66—C65—H65	119.0
O17—Sm2—O19	126.05 (19)	C64—C65—H65	119.0
O28—Sm2—O19	79.2 (2)	O6—C66—C65	127.5 (6)
O26—Sm2—O19	73.5 (2)	O6—C66—C67	113.4 (6)
O20—Sm2—O19	73.06 (19)	C65—C66—C67	119.2 (7)
O24—Sm2—O19	124.63 (16)	O5—C67—C62	122.6 (6)
O21—Sm2—O19	115.52 (19)	O5—C67—C66	118.4 (7)
O18—Sm2—O16	131.36 (15)	C62—C67—C66	119.0 (7)
O17—Sm2—O16	62.19 (17)	O6—C68—H68A	109.5
O28—Sm2—O16	75.9 (2)	O6—C68—H68B	109.5
O26—Sm2—O16	76.33 (19)	H68A—C68—H68B	109.5
O20—Sm2—O16	133.2 (2)	O6—C68—H68C	109.5
O24—Sm2—O16	65.47 (17)	H68A—C68—H68C	109.5
O21—Sm2—O16	95.62 (18)	H68B—C68—H68C	109.5
O19—Sm2—O16	148.86 (17)	O19—C69—H69A	109.5
O18—Sm2—O23	134.04 (16)	O19—C69—H69B	109.5
O17—Sm2—O23	154.52 (18)	H69A—C69—H69B	109.5
O28—Sm2—O23	114.47 (17)	O19—C69—H69C	109.5
O26—Sm2—O23	64.02 (15)	H69A—C69—H69C	109.5
O20—Sm2—O23	68.97 (16)	H69B—C69—H69C	109.5
O24—Sm2—O23	48.99 (15)	C71—C70—O19	125.9 (7)
O21—Sm2—O23	103.15 (15)	C71—C70—C75	122.1 (8)
O19—Sm2—O23	79.12 (16)	O19—C70—C75	112.0 (6)
O16—Sm2—O23	94.47 (15)	C70—C71—C72	119.6 (7)
O18—Sm2—N36	104.7 (2)	C70—C71—H71	120.2
O17—Sm2—N36	93.42 (16)	C72—C71—H71	120.2
O28—Sm2—N36	25.34 (18)	C73—C72—C71	120.5 (7)
O26—Sm2—N36	25.28 (15)	C73—C72—H72	119.7
O20—Sm2—N36	144.42 (17)	C71—C72—H72	119.7
O24—Sm2—N36	115.51 (18)	C72—C73—C74	120.0 (8)
O21—Sm2—N36	164.57 (15)	C72—C73—H73	120.0
O19—Sm2—N36	75.45 (19)	C74—C73—H73	120.0
O16—Sm2—N36	74.01 (19)	C73—C74—C75	121.0 (6)
O23—Sm2—N36	89.26 (15)	C73—C74—C76	120.8 (6)
O18—Sm2—N34	71.2 (2)	C75—C74—C76	118.2 (6)
O17—Sm2—N34	94.85 (16)	O18—C75—C70	120.9 (7)
O28—Sm2—N34	155.5 (2)	O18—C75—C74	122.4 (6)
O26—Sm2—N34	149.31 (16)	C70—C75—C74	116.7 (7)
O20—Sm2—N34	25.05 (18)	N38—C76—C74	122.6 (6)
O24—Sm2—N34	68.9 (2)	N38—C76—H76	118.7
O21—Sm2—N34	25.66 (18)	C74—C76—H76	118.7
O19—Sm2—N34	93.5 (2)	N38—C77—C78	112.2 (4)
O16—Sm2—N34	116.7 (2)	N38—C77—C84	109.1 (4)
O23—Sm2—N34	86.55 (16)	C78—C77—C84	113.6 (5)
N36—Sm2—N34	168.7 (2)	N38—C77—H77	107.2
C40—O3—C39	117.1 (6)	C78—C77—H77	107.2
C40—O3—Sm1	116.5 (4)	C84—C77—H77	107.2
C39—O3—Sm1	123.2 (4)	C79—C78—C83	118.8 (7)
C45—O4—Sm1	124.4 (4)	C79—C78—C77	118.0 (6)
C67—O5—Sm1	127.2 (4)	C83—C78—C77	123.1 (6)
C66—O6—C68	117.2 (6)	C78—C79—C80	121.5 (8)
C66—O6—Sm1	117.9 (4)	C78—C79—H79	119.2
C68—O6—Sm1	124.9 (5)	C80—C79—H79	119.2
N31—O7—Sm1	96.3 (5)	C81—C80—C79	119.8 (8)
N31—O9—Sm1	97.3 (4)	C81—C80—H80	120.1
N30—O10—Sm1	94.0 (4)	C79—C80—H80	120.1
N30—O11—Sm1	100.6 (5)	C80—C81—C82	118.6 (8)
N29—O13—Sm1	95.7 (4)	C80—C81—H81	120.7
N29—O14—Sm1	97.9 (4)	C82—C81—H81	120.7
C96—O16—C98	117.5 (7)	C83—C82—C81	121.0 (9)
C96—O16—Sm2	113.8 (4)	C83—C82—H82	128 (5)
C98—O16—Sm2	125.3 (4)	C81—C82—H82	110 (5)
C97—O17—Sm2	121.5 (4)	C82—C83—C78	120.2 (8)
C75—O18—Sm2	126.3 (4)	C82—C83—H83	119.9
C70—O19—C69	116.4 (6)	C78—C83—H83	119.9
C70—O19—Sm2	117.0 (4)	N37—C84—C85	113.1 (5)
C69—O19—Sm2	126.5 (5)	N37—C84—C77	108.5 (4)
N34—O20—Sm2	98.0 (4)	C85—C84—C77	109.8 (5)
N34—O21—Sm2	95.5 (4)	N37—C84—H84	108.4
N35—O23—Sm2	95.2 (4)	C85—C84—H84	108.4
N35—O24—Sm2	99.2 (4)	C77—C84—H84	108.4
N36—O26—Sm2	96.8 (4)	C87—C86—C85	120.2 (6)
N36—O28—Sm2	97.6 (4)	C87—C86—H86	119.9
O15—N29—O13	123.1 (8)	C85—C86—H86	119.9
O15—N29—O14	121.5 (8)	C90—C85—C86	118.7 (6)
O13—N29—O14	115.3 (6)	C90—C85—C84	122.6 (6)
O15—N29—Sm1	174.1 (8)	C86—C85—C84	118.6 (5)
O13—N29—Sm1	59.3 (3)	C88—C87—C86	122.1 (8)
O14—N29—Sm1	56.6 (3)	C88—C87—H87	119.0
O12—N30—O11	121.7 (7)	C86—C87—H87	119.0
O12—N30—O10	122.2 (6)	C88—C89—C90	121.3 (8)
O11—N30—O10	116.1 (6)	C88—C89—H89	119.4
O8—N31—O7	122.2 (8)	C90—C89—H89	119.4
O8—N31—O9	122.3 (7)	C87—C88—C89	118.5 (7)
O7—N31—O9	115.5 (6)	C87—C88—H88	120.8
O8—N31—Sm1	178.3 (7)	C89—C88—H88	120.8
O7—N31—Sm1	58.3 (4)	C85—C90—C89	118.9 (8)
O9—N31—Sm1	57.2 (3)	C85—C90—H90	120.5
C61—N32—C54	123.4 (5)	C89—C90—H90	120.5
C61—N32—H32	114.0	N37—C91—C92	122.8 (6)
C54—N32—H32	121.5	N37—C91—H91	118.6
C46—N33—C47	124.8 (5)	C92—C91—H91	118.6
C46—N33—H33	119.8	C97—C92—C93	119.3 (7)
C47—N33—H33	114.6	C97—C92—C91	120.1 (6)
O22—N34—O20	122.0 (8)	C93—C92—C91	120.6 (6)
O22—N34—O21	122.3 (8)	C94—C93—C92	120.5 (7)
O20—N34—O21	115.7 (6)	C94—C93—H93	119.7
O22—N34—Sm2	173.1 (8)	C92—C93—H93	119.7
O20—N34—Sm2	57.0 (3)	C93—C94—C95	120.7 (7)
O21—N34—Sm2	58.9 (3)	C93—C94—H94	119.7
O25—N35—O23	123.2 (5)	C95—C94—H94	119.7
O25—N35—O24	120.2 (6)	C96—C95—C94	119.5 (7)
O23—N35—O24	116.6 (6)	C96—C95—H95	120.3
O27—N36—O26	121.3 (6)	C94—C95—H95	120.3
O27—N36—O28	123.8 (7)	C95—C96—O16	126.2 (7)
O26—N36—O28	114.9 (6)	C95—C96—C97	121.6 (6)
O27—N36—Sm2	178.5 (7)	O16—C96—C97	112.3 (6)
O26—N36—Sm2	57.9 (3)	O17—C97—C96	119.9 (6)
O28—N36—Sm2	57.0 (4)	O17—C97—C92	121.7 (6)
C91—N37—C84	125.5 (5)	C96—C97—C92	118.4 (6)
C91—N37—H37	107.3	O16—C98—H98A	109.5
C84—N37—H37	122.8	O16—C98—H98B	109.5
C76—N38—C77	125.8 (5)	H98A—C98—H98B	109.5
C76—N38—H38	119.5	O16—C98—H98C	109.5
C77—N38—H38	114.7	H98A—C98—H98C	109.5
O3—C39—H39A	109.5	H98B—C98—H98C	109.5

### Hydrogen-bond geometry (Å, º)

**Table:**

*D*—H···*A*	*D*—H	H···*A*	*D*···*A*	*D*—H···*A*
C98—H98*A*···O9^i^	0.98	2.58	3.419 (9)	144
C91—H91···O27^ii^	0.95	2.59	3.097 (8)	114
C91—H91···O12^iii^	0.95	2.33	3.227 (8)	156
C77—H77···O24^iv^	1.00	2.29	3.264 (8)	164
C76—H76···O21^iv^	0.95	2.50	3.399 (9)	158
C69—H69*A*···O14^v^	0.98	2.44	3.323 (10)	150
C68—H68*A*···O10	0.98	2.55	3.214 (11)	125
C68—H68*A*···O9	0.98	2.66	3.224 (11)	117
C65—H65···O20^vi^	0.95	2.64	3.485 (8)	148
C61—H61···O13^vi^	0.95	2.49	3.429 (8)	172
C54—H54···O11^vi^	1.00	2.30	3.277 (8)	165
C46—H46···O25	0.95	2.32	3.211 (8)	155
C46—H46···O8^i^	0.95	2.56	3.054 (8)	113
C39—H39*A*···O27^i^	0.98	2.54	3.338 (9)	138
N38—H38···O18	0.86	1.87	2.550 (6)	135
N33—H33···O4	0.86	1.87	2.545 (7)	134
N37—H37···O17	0.83	1.89	2.582 (7)	139
N32—H32···O5	1.04	1.71	2.578 (6)	138

Symmetry codes: (i) −*x*+1, *y*, −*z*+1; (ii) −*x*+1, *y*, −*z*; (iii) *x*, *y*, *z*−1; (iv) −*x*+1/2, *y*−1/2, −*z*; (v) −*x*+1/2, *y*−1/2, −*z*+1; (vi) −*x*+1/2, *y*+1/2, −*z*+1.
